# Production of uncommon carotenoids and lipids by red yeasts utilizing agri-food residues and waste cooking oil

**DOI:** 10.1007/s00253-025-13680-2

**Published:** 2026-01-20

**Authors:** Silvia Donzella, Diego Romano, Francesco Molinari, Sebastián Bermúdez Puga, Ricardo Pinheiro de Souza Oliveira, Concetta Compagno

**Affiliations:** 1 Department of Food, Environmental and Nutritional Sciences (DeFENS), University of Milan, Via L. Mangiagalli 25, 20133 Milan, Italy; 2 Department of Biochemical-Pharmaceutical Technology, Faculty of Pharmaceutical Sciences, University of São Paulo (USP), Avenida Professor Lineu Prestes, São Paulo, 580 Brazil

**Keywords:** Carotenoids, *Rhodotorula paludigena*, Microbial oil, Waste cooking oil, Sustainable fermentations

## Abstract

**Abstract:**

In recent years, the biotechnological production of carotenoids and lipids by yeasts emerges as a valuable strategy at the industrial level, also fitting the circular economy pillars when agri-food waste can be used as the main components of the culture media. In this study, bioprocesses employing red yeasts were developed using cost-effective agri-industrial residues, such as soy okara (a soybean industry byproduct). This low-cost substrate was investigated as a source of carbohydrates and essential nutrients, with its enzymatic pre-treatment optimized to create a balanced and efficient fermentation medium. The screening of a collection of red oleaginous yeasts identified *Rhodotorula paludigena* CBS 6565 and *Rhodotorula diobovata* CBS 324 as promising strains capable of efficiently producing both lipids and uncommon carotenoids. These two strains were cultivated in a lignocellulose hydrolysate-based medium supplemented with urea, validating the promising results of the screening. Urea, a cost-effective nitrogen source, was found to enhance carotenoid production compared to ammonium sulfate. Finally, soy okara was used as the fermentation medium for *Rhodotorula paludigena* CBS 6565. Soy okara underwent optimized enzymatic hydrolysis to maximize fermentable sugar release, while the addition of waste cooking oil and syrup from candied fruit processing significantly boosted carotenoid production, reaching 262.4 mg/L in 90 h. Among these, β-carotene and torularhodin contributed 140 mg/L and 72.5 mg/L, respectively. Furthermore, the yeast cells accumulated lipids, constituting 56% of their dry weight, with a final concentration of 18 g/L. Overall, this study underscores the synergy between agri-food waste valorization and the sustainable production of yeast biomass enriched in carotenoids and lipids, offering a versatile and high-value resource for various industrial applications.

**Key points:**

• *β-carotene, torulene, and torularhodin levels are highly strain-dependent in yeasts*

• *R. paludigena CBS 6565 achieved high carotenoid–lipid co-production yields*

• *Fed-batch with multiple agri-food residues enabled a sustainable bioprocess design*

**Supplementary Information:**

The online version contains supplementary material available at 10.1007/s00253-025-13680-2.

## Introduction

Carotenoids are high-value products with an expanding global market, expected to reach USD 2.1 billion by 2030, growing at about 3.5% from 2022 to 2030 (Global Carotenoids Market Size, Trends, Share, Forecast 2030). The increasing demand for carotenoids presents significant business opportunities. Besides being used as colorants, carotenoids have several biological benefits. They are powerful antioxidants that strengthen the immune system and have antimicrobial properties (Chew and Park 2004; Cardoso et al. 2017; Toti et al. 2018). β-Carotene, a source of vitamin A, has bioactive properties that help reduce the risk of diseases like cardiovascular disorders, muscular degeneration, and certain cancers (Grune et al. 2010).

Although chemical synthesis meets 80–90% of the market demand, there is a global trend toward increasing the use of natural carotenoids, especially those from renewable sources, in line with the circular economy (Ncube et al. 2023). While carotenoids like β-carotene, astaxanthin, and zeaxanthin can be extracted from plants, the yield is low due to their low concentration and complex extraction procedures (Adadi et al. 2018). Additionally, their availability and extraction depends on seasonality, geography, and potential competition with food production. Growing interest in sustainability is driving research into bioprocesses that upcycle waste and manage resources efficiently. Microbial production offers a promising alternative, with certain yeast species, such as *Xanthophyllomyces dendrorhous* and *Phaffia rhodozyma* (Domínguez-Bocanegra et al. 2007; Gassel et al. 2014; Stoklosa et al. 2019; Harith et al. 2020), being widely studied for carotenoid production. Other yeast genera like *Rhodosporidium*, *Rhodotorula*, *Sporobolomyces*, and *Sporidiobolus*, known as “red yeasts,” also produce carotenoids (Mannazzu et al. 2015; Watcharawipas and Runguphan 2023; Sereti et al. 2023). Besides β-carotene, some of these yeasts also produce torulene and torularhodin (Kot et al. 2018). In recent years, interest in producing rare carotenoids like torulene and torularhodin has rapidly increased, particularly for their potential nutritional benefits (Kot et al. 2018). Torulene (3′,4′-didehydro–β,γ-carotene) is a red carotenoid with higher antioxidant activity than β-carotene (Zoz et al. 2015). Torularhodin is a xanthophyll, one of the few carotenoids with a carboxylic acid function, and shows considerable antioxidant activity (Sakaki et al. 2002). It is particularly interesting because the presence of a carboxyl group enhances its solubility, suitable to be used as an additive in aqueous feed formulations (Zoz et al. 2015).

Some red yeast species are oleaginous, meaning they can produce and store large amounts of lipids, up to 70% of their dry weight (Ratledge 2013). Carotenoids produced by these yeasts are stored in lipid bodies, which protect them from degradation caused by oxygen or light, preserving their nutritional and biological benefits (Mezzomo and Ferreira 2016). However, extracting carotenoids from yeast cells is complex and inefficient (Valduga et al. 2009; Naz et al. 2023). One solution is using freeze-dried yeast cells as additives, since carotenoids are more bioavailable when dissolved in oils (Nagao et al. 2013; Papapostolou et al. 2023). The fatty acid composition of yeast-derived oils is like that of plant-based oils, with palmitic, stearic, oleic, linoleic, and linolenic acids, making them suitable for food applications (Capusoni et al. 2017). Carotenoid-rich microbial oils can be used in new food formulations, such as edible films, which can improve shelf life and nutritional value (Papadaki et al. 2019, 2023).

Many oleaginous “red yeasts” can efficiently metabolize a variety of carbon sources, making them ideal for producing bio-products from waste and residues (Galafassi et al. 2012; Athenaki et al. 2018; Kot et al. 2019; Donzella et al. 2021). Carotenoid production by yeasts is a valuable industrial strategy in line with the circular economy concept. This involves screening potential strains for their ability to use low-cost substrates and developing efficient bioprocesses to maximize yield, such as optimizing culture media and fermentation parameters (Frengova and Beshkova 2009; Carlos Mata-Gómez et al. 2014; Bertacchi et al. 2020; Silva Igreja et al. 2021). The waste composition is important for preparing culture media that enhance both carotenoid and lipid production. The effects of carbon, nitrogen, and inorganic salts in the fermentation medium for carotenoid production can vary depending on the yeast strains (El-Banna et al. 2012; Shariati et al. 2019; Kot et al. 2019). Generally, high carotenoid and lipid production occurs when the C/N ratio is high due to a higher concentration of sugar and nitrogen starvation (Braunwald et al. 2013). Additionally, the carotenoid profile depends not only on the strain but also on medium composition and culture conditions (Buzzini et al. 2007; Kot et al. 2018).

The purpose of this study was to evaluate carotenoid and lipid production and their temporal dynamics by red yeasts cultivated on agri-food residues. Specifically, the work compared the effect on product formation of ammonium sulfate and urea, a low-cost nitrogen source. A residue-integrated process based on enzymatically hydrolyzed soy okara with waste cooking oil supplementation to enhance acetyl-CoA availability was developed in fed-batch mode with a high C/N ratio, obtained by supplementing an agri-waste syrup.

## Materials and methods

### Strains, media, and growth conditions

The yeast strains used in this work are:

- RPLCB1 *Rhodotorula paludigena* CBS 6565
- RPLCB2 *Rhodotorula diobovata* CBS 324
- Mo38 *Rhodosporidium diobovatum* UBOCC-A-208033 (Burgaud et al. 2010)
- RGRDP3 *Rhodosporidiobolus azoricus* DBVPG 4620 (from yeast collection at University of Perugia)
- PTAPK4 *Rhodosporidiobolus azoricus* (Donzella et al. 2019)
- Mo35 *Rhodotorula mucilaginosa* UBOCC-A-208030 (Burgaud et al. 2010)
- Ex7 *Rhodotorula mucilaginosa* UBOCC-A-208010 (Burgaud et al. 2010)
- Mim146 *Rhodotorula rubra* (Facchetti et al. 2015)
- RGNR1 *Rhodotorula glutinis* NRRL Y 842
- RGRDP2 *Rhodotorula graminis* DBVPG7021 (from yeast collection at University of Perugia)

For long-term storage, yeast strains were maintained at − 80 °C on 15% (v/v) glycerol.

Yeast Peptone Dextrose (YPD) medium contained 10 g/L yeast extract (Biolife, Italy), 20 g/L peptone (Biolife, Italy), and 20 g/L glucose (Sigma-Aldrich, Italy). Yeast cells were cultivated at 28 °C in a rotary shaker at 150 rpm in baffled flasks with an air–liquid ratio of 5:1. YNB plates contained 20 g/L glucose, Yeast Nitrogen Base without amino acids (Difco BD, Italy), 0.17 g/L ammonium sulfate, 0.1 M MES hydrate (Sigma-Aldrich, Italy) to maintain pH 6, and 15 g/L agarose. Cells from pre-cultures (inoculated from glycerol stocks or Petri dish) grown overnight on YPD were harvested by centrifugation (5000 rpm/2300 rcf) for 10 min in Eppendorf 5415D centrifuge) and inoculated at OD660 0.1 in YNB medium or at OD_660_ 0.2 in soy okara hydrolysate–based medium. Cell growth was monitored by measuring the increase of optical density at 660 nm (OD_660_) using a spectrophotometer (Eppendorf, Italy).

### Preparation of soy okara hydrolysate

Dried soy okara (kindly provided from local farm) was stored at 4 °C in a tightly closed jar.

Small scale (2 mL) enzymatic hydrolysis was carried out by suspending the okara powder with a loading of 12% (w/v) in distilled water. The mixture was then sterilized in an autoclave (Cavallo S.r.l., Milan, Italy) at 0.5 atm, 112 °C for 30 min. After cooling to 50 °C, the mixture was adjusted to pH 5.5 added with different amounts (5, 10, 20 μL/mL) of Cellic CTec2 enzyme cocktail (hydrolytic activity > 1150 U/mL) (SAE0020, Sigma-Aldrich, Italy) and incubated at room temperature under magnetic stirring. After 24 h of hydrolysis, the sugar content was analyzed by enzymatic assay.

The same procedure was applied to test α-amylase from *Aspergillus oryzae* (A8220, specific activity ≥ 800 FAU/g, Sigma-Aldrich, Italy) on soy okara waste. Different amounts of α-amylase (10, 20 µL/mL) were added together with Cellic CTec2 enzyme cocktail and incubated at room temperature under magnetic stirring. Glucose content was analyzed by enzymatic assay after 24 h of digestion.

The higher scale hydrolysis was carried out in a 2-L bioreactor (Applikon Biotechnology, The Netherlands). The bioreactor filled with the reaction mixture (800 mL) was autoclaved for 1 h at 1 atm, 121 °C. Temperature was set at 30 °C, agitation at 650 rpm, and pH at 5.5. The hydrolysis was started by the addition of enzymes (1.46 mL of Cellic CTec2). After 24 h, the hydrolysate was centrifuged in sterile tubes at 5000 rpm/3214 rcf in Eppendorf 5804R centrifuge for 1 h, and the liquid phase stored at − 20 °C.

### Fed-batch cultivation

Fed-batch cultures were performed in a 2-L bioreactor (Applikon Biotechnology, The Netherlands), with a starting volume of 800 mL. Soy okara hydrolysate was sterilized by autoclaving at 112 °C (0.5 atm) for 30 min. Temperature was set at 28 °C and the air inlet at 1 vvm. The pH, measured by AppliSens pH electrode (Applikon Biotechnology, The Netherlands), was automatically adjusted and maintained at 6 by adding 5 M KOH or 10% (v/v) solution of H_2_SO_4_. Dissolved oxygen concentration was measured by AppliSens oxygen probe (Applikon Biotechnology, The Netherlands), starting from 100% of saturation. The cascade mode was set to maintain oxygen over 30% with stirring ranging from 400 to 800 rpm. Foam formation was controlled by the addition of a silicon antifoaming agent (Sigma 204 from Sigma-Aldrich, Italy). Sterilized and diluted syrup from candied fruits (mango) manufacture (SVZ, Industrial Fruit & Vegetable Ingredients, Breda, the Netherlands), containing glucose 199 g/L and fructose 296 g/L, was supplied after 46 h as carbon feed.

### Sugars determination

The concentrations of sugars during enzymatic treatments and fermentation processes were determined by employing commercial enzymatic kits (K-GLUHK, K-SUFRG, K-XYLOSE, K-LACGAR from Megazyme, Ireland). All the assays were performed in triplicate and standard deviations varied between 1 and 5%.

### Nitrogen determination

Inorganic nitrogen was determined by employing a commercial enzymatic kit (10542946035, R-Biopharm AG, Germany). Total nitrogen concentration in culture supernatants was determined by Kjeldahl method using a SpeedDigester K-376 and a KjelMaster K-375 (Buchi, Italy).

### Dry weight determination

Cells were collected from the medium (1 or 2 mL of cell culture) by centrifugation (5 min at 13,200 rpm/16,100 rcf in Eppendorf 5415D centrifuge). The pellets were dried overnight at 105 °C. In the case of soy okara, the dry weight of the pellet at T0 was determined and subtracted from the value of the following measurements. The biomass and product yields were calculated as the ratio between the total amount of biomass or products and the amount of consumed sugars.

### Total lipid quantification

Lipid content was determined via the sulfo-phospho-vanillin (SPV) colorimetric method (Spinreact, Spain) on the washed cell pellets corresponding to approximately 30 OD, suspended in 0.5 mL of cold redistilled water. The assays were performed in triplicate, and standard deviations varied between 1 and 5%. Lipid yield was calculated as the ratio between the total amount of product and the amount of consumed carbon sources.

### Carotenoid extraction and quantification

The fermented broth (0.5 mL) was collected in screw cap tubes and centrifuged at 13,200 rpm/16,100 rcf for 10 min, and the cell pellet was washed with distilled water. Five hundred microliters of hexane:ethylacetate mixture (1:1, v/v) and an equivalent volume (500 µL) of glass beads (425–600 μm from Sigma) were added to the pellet. Other tested solvents included: acetone, 100% phosphate buffer, DMSO. Each sample underwent five cycles in a bead beater (Bertin Technologies, France) at 4 °C with 1-min resting intervals until the pellet became colorless. Alternatively, multiple vortexing with pauses in ice was tested but the pellet never became completely colorless.

The resulting pink/red liquid was dried under nitrogen flow and resuspended in the mobile phase used for HPLC quantification. Carotenoids were quantified using high-performance liquid chromatography (HPLC) (Merck Hitachi) equipped with a C18 column (RP-18e) and a UV detector at a wavelength of 457 nm. The mobile phase consisted of acetonitrile, hexane, and ethylacetate (4:4:2, v/v/v) at a flow rate of 0.7 mL/min. The carotenoid concentrations were quantified using standard calibration curves of β-carotene (Sigma-Aldrich, Italy, retention time 11 min), torulene (CaroteNature, Switzerland, retention time 7.5 min), and torularhodin (CaroteNature, Switzerland, retention time 5.5 min).

### Yield and productivity calculation

Carotenoid production performance was assessed by calculating the yield on substrate (*Y*_P/S_) and volumetric productivity (QP) as reported in Malisorn and Suntornsuk (2009). Carotenoid yield on substrate (mg/g) was reported as mg of carotenoid (quantified by HPLC) per g of sugars consumed (quantified by enzymatic kits). The volumetric productivity (expressed as mg/L/h) was calculated by dividing the final carotenoid concentration by the cultivation time.

## Results

### Screening of red yeasts for carotenoid production and optimization of their extraction

**Fig. 1:**
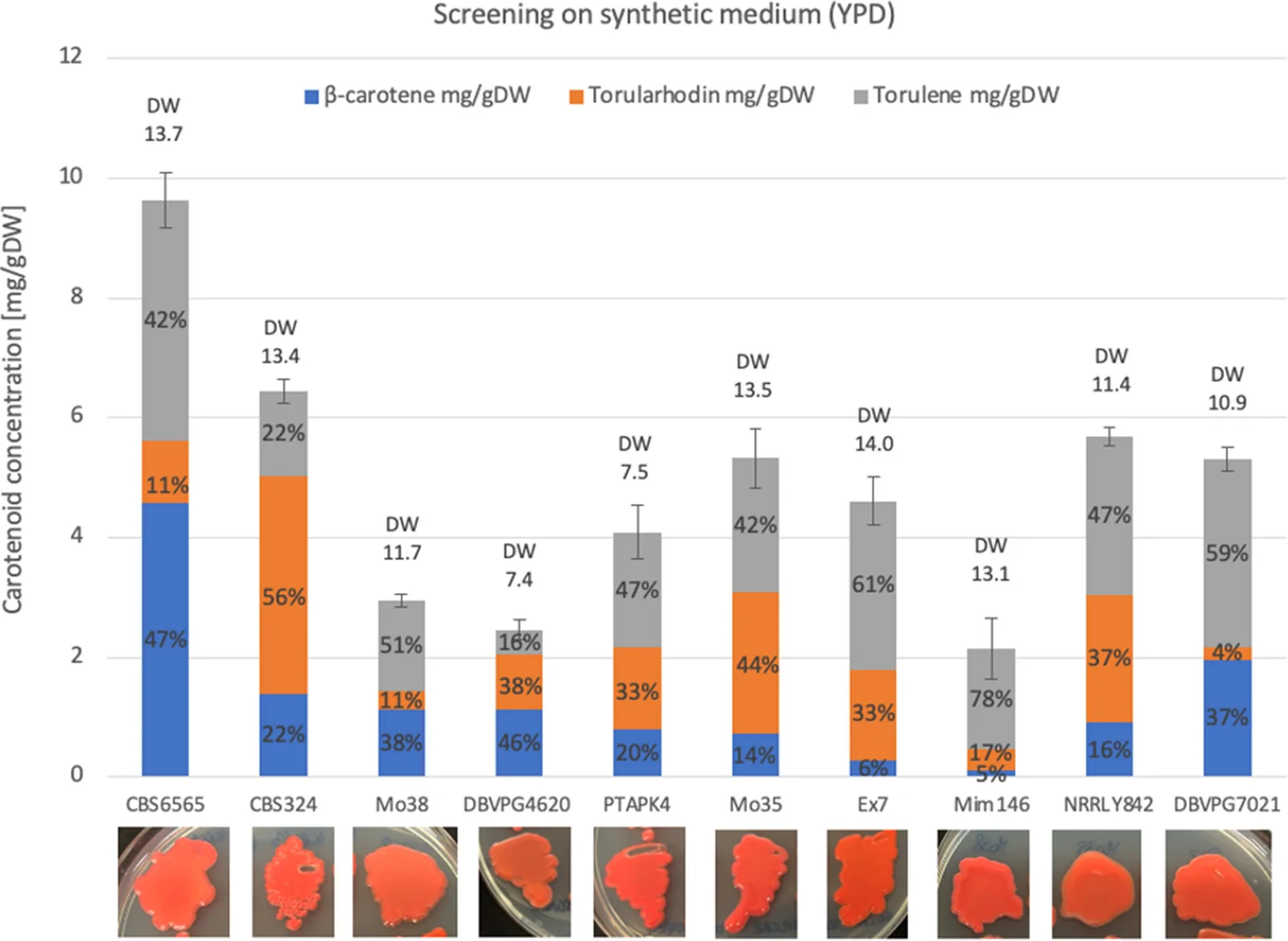
Screening of carotenoid content and its profile (β-carotene in blue, torularhodin in orange, and torulene in grey) after 48 h of growth in YPD. Labels above columns refer to cell dry weight (DW, g/L) of the sample. Labels inside columns refer to the percentage distribution of the specific carotenoid of the total. Photos were taken after 48 h of growth on YNB plates at 28 °C

A screening of 10 red oleaginous strains from *Rhodotorula/Rhodosporidium* species was performed in YPD medium to evaluate their ability to produce β-carotene, torularhodin, and torulene. This screening was performed in Yeast Peptone Dextrose (YPD), a nutrient-rich synthetic medium that supports rapid growth, but generally is not suitable for lipid accumulation. After 48 h of growth, cells were collected and analyzed for biomass (dry weight, DW) and carotenoid content (mg/gDW). To enable a more meaningful comparison among the different strains, the carotenoid production was expressed as carotenoid content thus normalized to DW (Fig. 1). Due to numerous extraction methods found in literature, various protocols were tested; the most effective involved bead beating with glass beads, enabling automated pigment release and avoiding degradation. Carotenoid composition was quantified by HPLC after building calibration curves using commercial standards as reference (see the “Materials and methods” section).

A significant variability in carotenoid composition was found across the strains. The amount of β-carotene was in the range of 0.1–4.6 mg/gDW, with strain CBS 6565 being the best producer (Fig. 1). Similarly, torularhodin levels varied considerably: while some strains exhibited negligible amounts (e.g., Mo38 and Mim 146), strain CBS 324 produced significant amounts (3.5 mg/gDW, 56% of the total carotenoids produced). Strain CBS 6565 resulted in the best producer in terms of both β-carotene (4.6 mg/gDW, corresponding to 102.70 mg/L) and torulene (4.0 mg/gDW, corresponding to 90.4 mg/L), after 48 h of growth.

Based on these results, *Rhodotorula paludigena* CBS 6565 and *Rhodotorula diobovata* CBS 324 showed the highest carotenoid content with different compositions and were thus selected for further studies.

### Production of carotenoids and lipids using glucose and xylose as mixed carbon sources

Lignocellulosic hydrolysates serve as an efficient medium for sustainable fermentation. To evaluate their effectiveness in lipid and carotenoid production by *Rhodotorula paludigena* CBS 6565 and *Rhodotorula diobovata* CBS 324, we tested a medium with glucose and xylose as mixed carbon sources in a 2:1 ratio, a composition typical of lignocellulosic hydrolysates.

These experiments also allowed the evaluation of the impact of different N-sources on fermentation, since its importance for both biomass and lipid synthesis (Shaigani et al. 2021). In particular, urea and ammonium sulfate were selected as widely available nitrogen sources with documented impacts on red-yeast physiology. Ammonium has been reported to support higher biomass and lipid titers under high C/N conditions, whereas information on the impact of urea is still limited (Braunwald et al. 2013; Peng et al. 2021; Sereti et al. 2024; Donzella et al. 2022). Considering the well-established influence of the nitrogen source on both lipid and carotenoid production, evaluating both compounds provided process-relevant guidance for cost-sensitive media design.

**Fig. 2:**
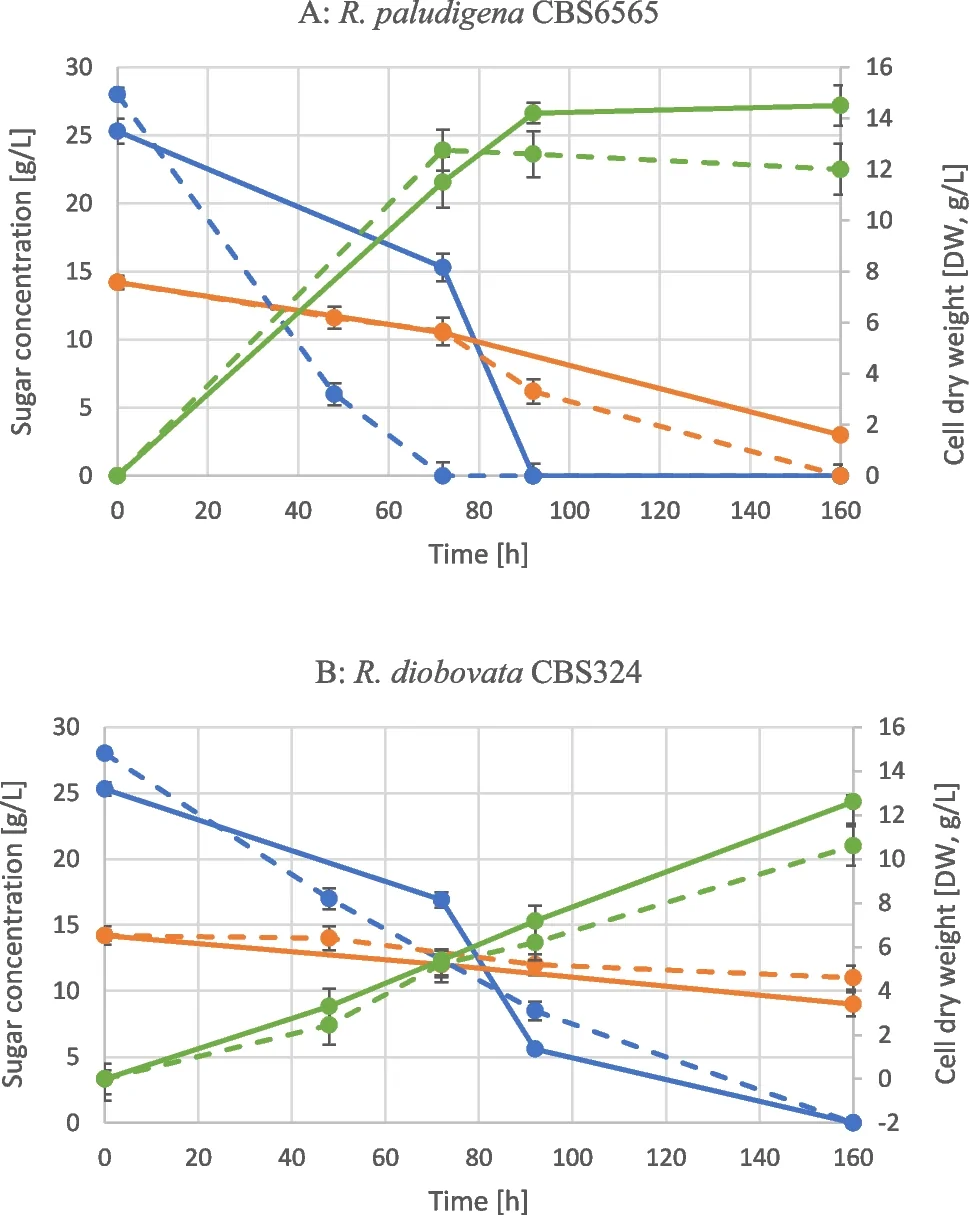
Sugar consumption and biomass production of *R. paludigena* CBS6565 (**A**) and *R. diobovata* CBS 324 (**B**) in the presence of urea (full lines) and (NH_4_)_2_SO_4_ (dotted lines). Blue line, residual glucose (g/L); orange line, residual xylose (g/L); green line, cell dry weight (DW, g/L)

**Fig. 3:**
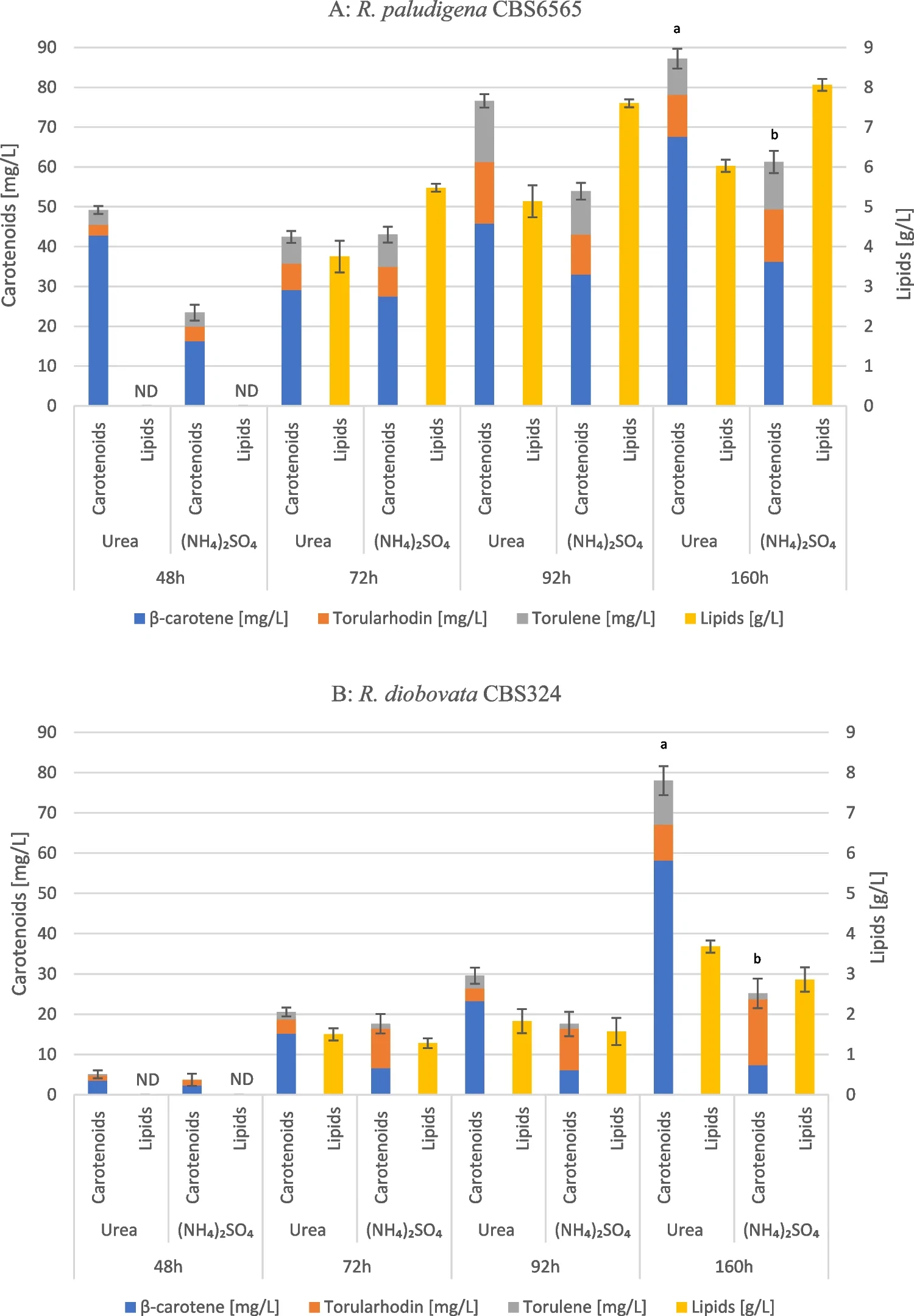
Carotenoid and lipid production of *R. paludigena* CBS 6565 (**A**) and *R. diobovata* CBS 324 (**B**) in the presence of urea or (NH_4_)_2_SO_4_. ND, not determined. Standard deviations are referred to total carotenoid concentration values. Data of total carotenoid concentrations at 160 h of both strains resulted in significant (a, b, *p*-value < 0.01) by one-way ANOVA with post hoc Tukey HSD

*Rhodotorula paludigena* CBS 6565 and *Rhodotorula diobovata* CBS 324 were cultured in a medium with a high carbon-to-nitrogen (C/N) ratio of 75 to promote lipid accumulation. *R. paludigena* CBS 6565 was found to be more efficient in metabolizing glucose and xylose when ammonium sulfate was used as an N-source (Fig. 2A), achieving higher biomass in 160 h (14.5 g_dry weight_/L in the presence of ammonium sulfate vs. 12.0 g_dry weight_/L with urea). Nevertheless, in the presence of urea, this strain produced 87 mg/L of total carotenoids, compared to 62 mg/L using ammonium sulfate as an N-source (Fig. 3A). When normalized to the substrate consumed, these titers corresponded to product yields (*Y*_P/S_) of 2.20 mg/g with urea and 1.44 mg/g with ammonium sulfate, clearly showing that urea promoted a more efficient conversion of sugars into carotenoids. In terms of volumetric productivity, the same cultivations reached 0.54 mg/L/h in the urea-supplemented medium and 0.39 mg/L/h in the ammonium-based medium, highlighting that urea not only increased the yield but also improved the overall rate of carotenoid formation. This difference was primarily due to a significant rise in β-carotene content, which resulted in 65 mg/L in the urea-supplied medium compared to 37 mg/L in the ammonium-supplied medium, in contrast to the levels of other carotenoids that were similar in both conditions. The lipid content accounted for approximately 50% of the dry weight in both conditions. However, the higher cell biomass yield in the ammonium sulfate medium led to increased overall lipid titers (Fig. 3A).

*R. diobovata* CBS 324 did not efficiently metabolize xylose that was always found at the end of the process (Fig. 2B). Lipid content was modest (Fig. 3B), being below 35% under both nitrogen conditions. Nevertheless, after 160 h of growth, the carotenoid accumulation in the presence of urea resulted in a yield similar to those of *R. paludigena* CBS 6565 (Fig. 3A, B). Specifically, *R. diobovata* attained 25 mg/L of total carotenoids in ammonium-based medium, corresponding to a product yield (*Y*_P/S_) of 0.58 mg/g, while it reached a concentration of 78 mg/L in the presence of urea, reaching a product yield of 1.97 mg/g. Interestingly, *R. diobovata* showed a distinctive carotenoid profile, with torularhodin representing a major fraction of the pigments.

Both red yeast species increased carotenoid production when grown on urea-based medium compared to ammonium sulfate, highlighting the suitability of this low-cost compound as a nitrogen source for maximizing carotenoid yield.

### Soy okara enzymatic pre-treatment and utilization as fermentation medium

Okara, a byproduct of soymilk and soybean curd manufacturing, is often discarded despite being rich in essential nutrients such as carbohydrates, proteins, lipids, minerals, and phytochemicals. In accordance with circular economy principles, soy okara was assessed as a sustainable and cost-effective feedstock for fermentation to produce carotenoids and lipids using red yeasts. To enhance the availability of fermentable sugars for yeast growth, the dry material underwent enzymatic hydrolysis. Based on a previous study (Donzella et al. 2021), a commercial enzyme blend containing cellulases, β-glucosidases, and hemicellulases (Cellic CTec2 from Sigma) was tested at different concentrations (5 to 20 μL/mL) to optimize the hydrolysis of soy okara. The enzymatic concentration of 10 μL/mL was the most effective, releasing 18.7 g/L of glucose (Table 1). Considering the possible presence of starch, the addition of commercial amylase (A8220 from *Aspergillus oryzae*), together with the Cellic CTec2 blend, was tested to check if it could increase the amount of glucose in the hydrolysate. The combination of the two commercial blends did not significantly increase the glucose release; thus, the use of only 10 μL/mL of Cellic CTec2 was selected as the best pre-treatment in terms of costs/yield (Table 1). The sugar composition of the hydrolysate was fully assessed, showing no presence of sucrose, galactose, or fructose, while containing a small amount of xylose (1 g/L). Total nitrogen analysis (Kjeldahl method) detected the presence of organic nitrogen in the range of 1–1.2 g/L; for this reason, no nitrogen source addition was needed since the estimated C/N ratio in this medium resulted below 20.

**Table 1:** Optimization of okara waste enzymatic pre-treatment

Enzyme/s	Enzyme concentration (μL/mL)	Glucose (g/L)
	Control (no enzymes)	2.85 ± 1.01
Cellic CTec2	5	11.18 ± 0.34
	10	18.71 ± 0.81
	20	17.13 ± 0.29
Cellic CTec2 + Amilase A8220	10 + 10	15.13 ± 1.10
	20 + 20	16.64 ± 0.97

**Fig. 4:**
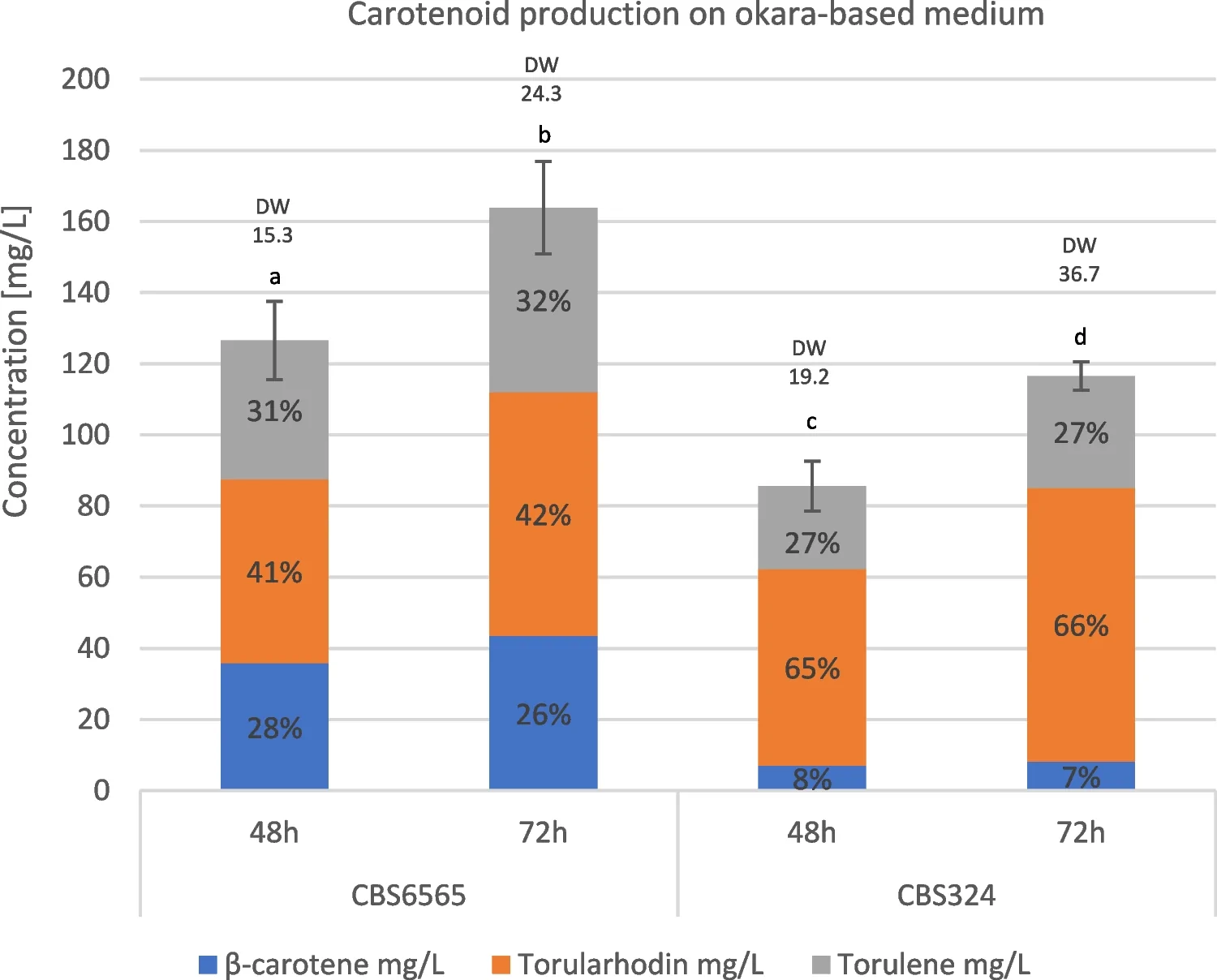
Carotenoid production after 72 h of process on okara-based medium with a feed of mango waste supplied at 48 h. DW, cell dry weight (g/L). All data of total carotenoid concentrations resulted significant (a, b, c, d, *p*-value < 0.01) by one-way ANOVA with post hoc Tukey HSD. Standard deviations are referred to total carotenoid concentration values

Fermentation with the two most promising strains using okara hydrolysate showed that after 48 h, both strains produced more biomass compared to the YPD medium (Fig. 4), likely due to the higher concentration of nitrogen sources and other nutrients within the okara hydrolysate. When evaluating β-carotene production at this timepoint, *R. paludigena* CBS 6565 resulted in the highest concentration both as mg/L (35.5 mg/L) and mg/gDW (2.35 mg/gDW). Overall, we noticed that okara-based medium boosted the production of torularhodin and torulene over β-carotene for all the tested strains.

However, after 48 h of growth, the glucose derived from soy okara hydrolysis was almost depleted by all yeasts, raising the need for a two-step process. The cultures were then fed with another waste residue, a syrup derived from mango candied processing already validated in similar bioprocesses (Donzella et al. 2021), which contains zero nitrogen but a high concentration of available glucose and fructose (199 and 296 g/L, respectively) without the need of any pre-treatment. After this addition, the biomass continued to increase, indicating the ability of all those strains to metabolize both sugars present in this feed. At the end of the fermentation process (72 h), *R. paludigena* CBS 6565 exhibited the highest β-carotene level (43.5 mg/L, Fig. 4), maintaining its lead. Torularhodin production was notably higher in *R. diobovata* CBS 324, reaching 77 mg/L (corresponding to 2.1 mg/gDW), the highest among all screened conditions, implying that *R. diobovata* CBS 324 is particularly efficient at synthesizing this carotenoid. Regarding torulene production, *R. paludigena* CBS 6565 again showed superior performance with the highest concentration, 51.7 mg/L and 2.2 mg/gDW. Additionally, lipid production at the end of the process under these conditions was assessed, yielding 10 g/L for *R. paludigena* CBS 6565 (41.1% of cell dry weight) and 14 g/L for *R. diobovata* CBS 324 (38.8% of cell dry weight). This was attributed to the syrup feed, which skewed the C/N ratio in favor of lipid synthesis.

For completeness, we also tested the performance of all strains in okara hydrolysate. However, the carotenoid production for these strains was lower compared to the two selected strains (data available in the supplementary materials, S1). In conclusion, *R. paludigena* CBS 6565 and *R. diobovata* CBS 324 consistently demonstrated high carotenoid production in waste-derived medium across the other strains, with the first exhibiting a significant carotenoid increase after 72 h of process. These strains resulted particularly effective in utilizing okara and mango syrup residues for carotenoid biosynthesis, biomass production, and lipid accumulation.

### Improving the fermentation process: adding waste cooking oil to increase production in a bioreactor

The process in okara medium employing the best producer strain (*R. paludigena* CBS 6565) was scaled up in a 2-L bioreactor under controlled conditions. The okara enzymatic treatment was performed directly in the bioreactor vessel for 24 h. Before inoculum, yeast extract (1 g/L) and waste cooking oil at 1% w/v were added to the hydrolysate to boost biomass production and acetyl-CoA availability, possibly increasing lipid and carotenoid synthesis.

The fermentation conditions were set with stirring between 400 and 800 rpm in cascade mode to keep the oxygen level above 30%. As observed in flask cultures, a two-step process was required. The mango syrup feed was applied after 46 h of growth, when sugars from okara waste were almost exhausted.

**Fig. 5:**
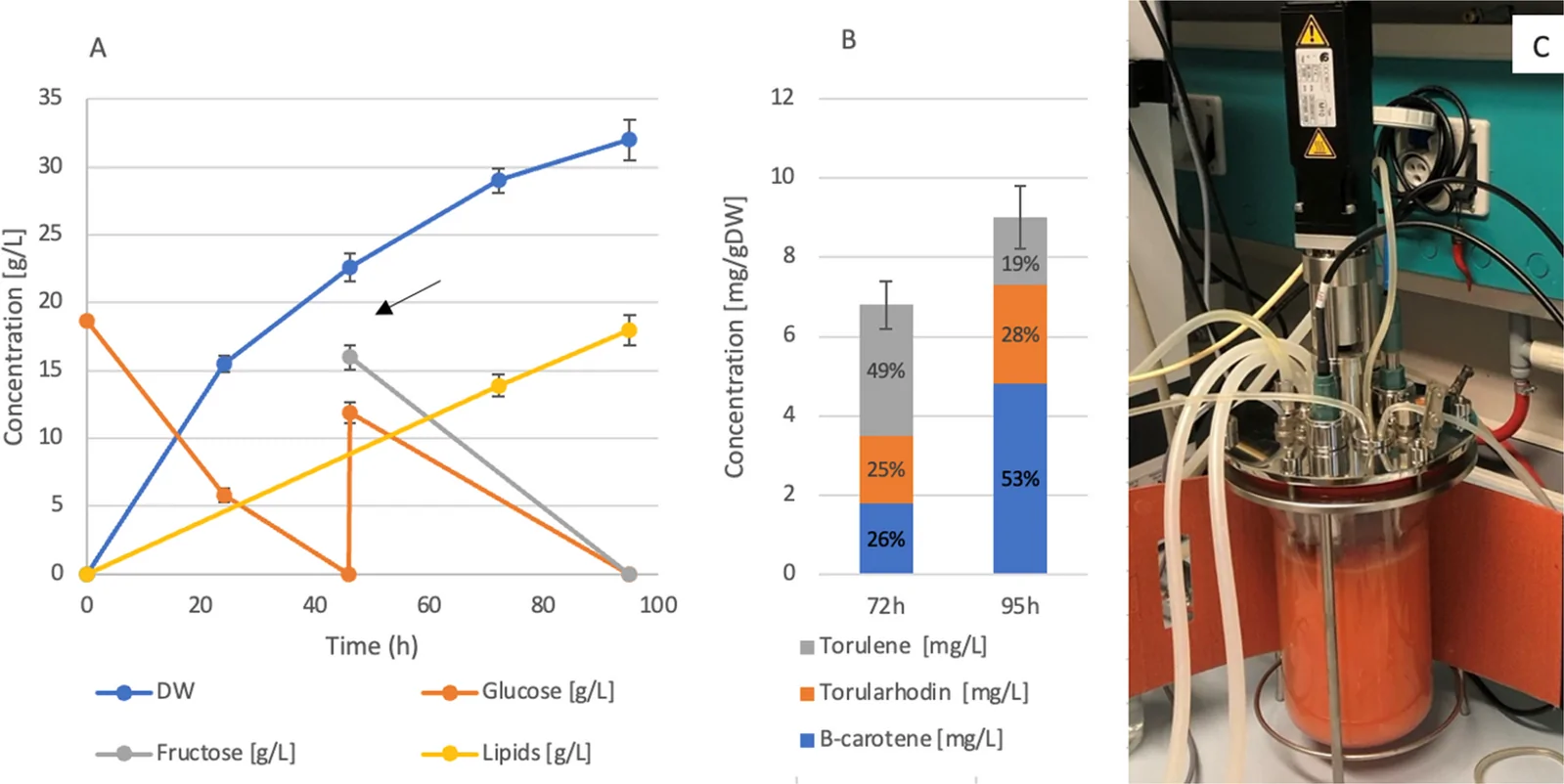
**A** Sugar consumption and production of biomass and lipids in bioreactor on okara-based medium supplemented with waste cooking oil. The arrow indicates the mango syrup feed. **B** Carotenoid production and profile in bioreactor on okara-based medium supplemented with waste cooking oil (1% w/v). **C** Photo of the bioreactor after mango syrup feed (48 h)

Compared to flask cultures, we observed an increase in both biomass and carotenoid content, corresponding to 189.3 mg/L (6.8 mg/gDW) of total carotenoids in 72 h (Fig. 5). At the end of the process (95 h), the pigment concentration still increased, achieving 262.4 mg/L (9 mg/gDW, Fig. 5B), but with a different composition, since torulene decreased while β-carotene and torularhodin, carotenoids characterized by more powerful antioxidant proprieties, almost doubled, reaching a concentration of 140 mg/L and 72.5 mg/L, respectively.

The lipid content was also measured, resulting in 18 g/L, or 56.2% of the cell dry weight, at the end of the process (Fig. 5A). This value matches previous results from other oleaginous species grown on agri-food residues (Donzella et al. 2021, 2022, 2024), demonstrating the effectiveness of soy okara as a fermentation feedstock for both carotenoid and lipid production.

In conclusion, these results support the growing concept of the “circular bioeconomy,” which focuses on sustainably using biomass in a zero-waste model, turning waste into a resource that supports industrial production.

## Discussion

To study carotenoid production in detail, it was first necessary to optimize the extraction protocols, confirming prior evidence of the key role played by cell wall disruption in pigment release. As previously reported (Saenge Chanika et al. 2011; Amado and Vázquez 2015; Zeng et al. 2023; Pham et al. 2024), the efficiency of carotenoid extraction from yeast cells is strongly dependent on the methodology employed. In agreement with these findings, our study demonstrated that using glass beads to break the cell wall significantly enhanced extraction yield compared to solvent-only methods (see the “Materials and methods” section). This effect was further amplified by substituting the vortex with a bead beater for pellet disruption, which enabled single-step solvent addition and full automation of the procedure. Multiple cooling cycles were included to prevent overheating and pigment degradation. The possibility of scaling and automating this method further underscores its potential for industrial application.

Our results also revealed a marked natural variability among *Rhodotorula* and *Rhodosporidium* strains, both in carotenoid composition and productivity. Notably, *R. paludigena* CBS 6565 outperformed all other strains in β-carotene and torulene production, while *R. diobovata* CBS 324 displayed a distinct torularhodin-rich profile (Fig. 1). These differences reflect species-specific metabolic pathways that can be selectively exploited in bioprocesses tailored to the desired pigment profile.

The composition of the fermentation medium emerged as a critical factor influencing microbial carotenogenesis. Both the type and nature of carbon and nitrogen sources played a decisive role in shaping metabolic outputs, and its understanding is crucial, especially with the perspective of employing waste feedstock as fermentation medium. Consistent with previous studies (Yimyoo et al. 2011; Peng et al. 2021; Sereti et al. 2024), we observed that both ammonium sulfate and urea supported biomass and pigment synthesis, but with different outcomes. Ammonium sulfate promoted higher biomass and lipid yields, whereas urea more effectively enhanced carotenoid accumulation (particularly β-carotene), suggesting nitrogen-dependent metabolic regulation at the cellular level.

With regard to carbon sourcing, the use of agri-industrial wastes such as okara and mango syrup proved highly effective, as previously demonstrated in fermentation processes for producing various functional ingredients and food products (Asghar et al. 2023; Donzella et al. 2022). Okara hydrolysate not only supported growth but also improved pigment diversity, promoting torularhodin and torulene over β-carotene (Fig. 4). Furthermore, the implementation of a two-step feeding strategy with mango syrup extended biomass growth and product formation over time, thereby improving overall productivity. From a practical point of view, the very high sugar concentration of the mango syrup makes it particularly effective as a feed, since small additions are sufficient to sustain the process without significantly altering the fermentation volume, while at the same time facilitating handling and long-term storage by reducing water activity and thereby minimizing the risk of microbial contamination.

Carotenoid biosynthesis was also strongly affected by fermentation conditions in the bioreactor. Several studies (Kim et al. 1997; Aksu and Tuǧba Eren 2005; Keskin et al. 2023; Donzella et al. 2024) have proposed oils, detergents, and surfactants as carbon-rich additives capable of stimulating both biomass and pigment production. Consistent with literature on oil-supplemented cultivations, adding waste cooking oil likely increased acetyl-CoA availability, supporting both lipid accumulation and the shift toward β-carotene/torularhodin. This strategy supports its use as a bwidely available residue, providing a realistic, low-cost intensification lever for waste-based fermentations. Given the strictly aerobic nature of carotenogenesis, dissolved oxygen levels were tightly controlled, and prolonged cultivation under oxygen-rich conditions (> 30%) led to changes in carotenoid profile. In particular, the marked increase in torularhodin concentration may be attributed to the oxidative conversion of torulene, in line with the biosynthetic pathway described by Kot et al. (2019), where torularhodin derives from torulene via hydroxylation and oxidation steps.

Benchmarking against prior works, the total carotenoid concentration achieved in this study through a fed-batch strategy—262.4 mg/L—using waste-based substrates is comparable with, or even exceeds, the highest β-carotene concentrations reported in optimized fermentations using pure carbon sources. For instance, *R. paludigena* CM33 was shown to reach 251.64 mg/L of β-carotene when cultivated in fed-batch using glucose, and up to 285.00 mg/L using sucrose under similar conditions but with enhanced feed concentrations (Thumkasem et al. 2024). These values also align with previous studies employing residue streams for red yeasts. For example, Aksu and Eren (2005) reported that *Rhodotorula glutinis* cultivated on molasses-based media produced up to 89 mg/L of carotenoids, showing the feasibility of sugar-rich residues for pigment production. Kot et al. (2019) demonstrated that glycerol and wastewater supported lipid accumulation of approximately 8–10 g/L and carotenoid titers of 6.5 mg/L. Attaining 262.4 mg/L of total carotenoids using low-cost agri-industrial waste streams represents a remarkable result, and the significant co-production of β-carotene (140 mg/L) and torularhodin (72.5 mg/L) is particularly valuable, providing a mixed product with diverse bioactivities (Sereti et al., 2024). Although β-carotene has a more established market, torularhodin is a stronger antioxidant than β-carotene and is currently under investigation for its potential. This compound is not yet commercially available, but its unique bioactivities and sustainable microbial production could open a new market for natural molecules with high-value applications (Sereti et al., 2024).

In addition to carotenoid production, the simultaneous accumulation of lipids at both high concentration (18 g/L) and high intracellular content (56%) represents a significant added value of the process, suggesting an efficient conversion of low-cost feedstocks into valuable cellular components. This dual production strategy provides a considerable economic advantage by enabling full valorisation of the yeast biomass, with potential applications in both pigment and bio-oil industries.

Overall, this study demonstrates the feasibility of integrating low-cost substrates and waste-derived feedstocks into high-yield, carotenoid-producing bioprocesses. The strains examined, particularly *R. paludigena* CBS 6565, showed strong potential for sustainable pigment biosynthesis using circular bioeconomy strategies, converting agricultural by-products into valuable compounds while reducing waste and cost.

## Conclusions

This work highlights the potential of *Rhodotorula paludigena* CBS 6565 and *Rhodotorula diobovata* CBS 324 for the sustainable co-production of carotenoids and lipids using agri-industrial residues. Enzymatically hydrolyzed soy okara provided a nutrient-rich, cost-effective medium, while the use of urea as a nitrogen source significantly enhanced carotenoid biosynthesis. The integration of additional waste-derived substrates, such as waste cooking oil and mango syrup, further boosted biomass and product yields in bioreactor-scale fermentations. Under optimized conditions, *R. paludigena* CBS 6565 achieved 262.4 mg/L of total carotenoids and 18 g/L of lipids, demonstrating the strain’s robustness and industrial potential. These findings support the development of circular bioeconomy strategies that valorize food industry by-products for the microbial production of high-value compounds.

## Supplementary Information

Below is the link to the electronic supplementary material.

ESM 1 (27.6 KB DOCX)

## Data Availability

No datasets were generated or analysed during the current study.
