# Supplementary material for: Production of uncommon carotenoids and lipids by red yeasts utilizing agri-food residues and waste cooking oil
Source: Appl Microbiol Biotechnol. 2026 Jan 20;110:29. doi: 10.1007/s00253-025-13680-2 (PMC12823764; doi:10.1007/s00253-025-13680-2)
Supplement: Supplementary file 1 [file 253_2025_13680_MOESM1_ESM.docx]

SUPPLEMENTARY MATERIALS

Carotenoid production on okara-based medium

Concentration [mg/L]

|  | β-carotene mg/L | Torularhodin mg/L | Torulene mg/L |
| --- | --- | --- | --- |
| Mo38 48h | 6.865 | 29.84 | 42.885 |
| Mo38 72h | 3.805 | 51.64 | 45.485 |
| DBVPG4620  48h | 5.965 | 22.315 | 37.805 |
| DBVPG4620  72h | 3.05 | 27.34 | 42.175 |
| Mo35 48h | 4.535 | 30.35 | 33.995 |
| Mo35 72h | 3.975 | 31.205 | 42.035 |
| Ex7 48h | 0.5 | 24.805 | 20.005 |
| Ex7 72h | 0.5 | 25 | 29.755 |

S1. Carotenoid production after 72h of process on okara based medium with a feed of mango waste supplied at 48h. DW: Cell dry weight (g/L).
