# Supplementary material for: Production of uncommon carotenoids and lipids by red yeasts utilizing agri-food residues and waste cooking oil
Source: Appl Microbiol Biotechnol. 2026 Jan 20;110(1):29. doi: 10.1007/s00253-025-13680-2 (PMC12823764; doi:10.1007/s00253-025-13680-2)
Supplement: Supplementary file 1 — (27.6 KB DOCX) [file 253_2025_13680_MOESM1_ESM.docx]

SUPPLEMENTARY MATERIALS

S1. Carotenoid production after 72h of process on okara based medium with a feed of mango waste supplied at 48h. DW: Cell dry weight (g/L).
